# The economic impact of porcine reproductive and respiratory syndrome outbreak in four Chinese farms: Based on cost and revenue analysis

**DOI:** 10.3389/fvets.2022.1024720

**Published:** 2022-10-12

**Authors:** Zhendong Zhang, Zhi Li, Hao Li, Shuqing Yang, Fubo Ren, Ting Bian, Liumei Sun, Bin Zhou, Lei Zhou, Xiangyang Qu

**Affiliations:** ^1^School of Biotechnology, Jiangsu University of Science and Technology, Zhenjiang, China; ^2^College of Veterinary Medicine, China Agricultural University, Beijing, China; ^3^Nanjing Dr. Vet Health Management Co., Ltd., Nanjing, China; ^4^College of Veterinary Medicine, Nanjing Agricultural University, Nanjing, China

**Keywords:** PRRS outbreak, PRRS control, economic impact, load-close-exposure, productive parameters

## Abstract

The economic impact after the outbreak of porcine reproductive and respiratory syndrome (PRRS) has been proven to be tremendous for pig production worldwide. However, the economic impact of the disease is not well understood in China. In our previous study, we acquired and analyzed the main production data (the number of weaned piglets, health costs, delayed marketing age, etc.) from the management system before and after the PRRS outbreaks occurring in November 2014, March 2015, December 2016, and February 2017. This study aimed to analyze and quantify the economic losses of the four PRRS outbreaks in Chinese herds. A straightforward approach was used to calculate additional costs and decreased revenues based on the PRRS-induced production deficiencies by average cost-of-production indices calculated from annual estimates of costs between 2014 and 2017. The results showed that economic losses varied between ¥668.14 and ¥1004.43 per sow in breeding herds from the outbreaks to regain the basic performance, with an average of ¥822.75 per sow, and the mean costs in the fattening herds (including nursery pigs) were ¥601.62 per sow, ranging from ¥318.64 to ¥937.14. Overall, the economic impact of PRRS on the whole herd was ¥1424.37 per sow. The majority of the losses were due to the reduction in the number of weaned piglets for breeding herds, and the increased feed cost (occupying 44.88%) was the primary source of loss for fattening herds. Our study fills the gap in knowledge of PRRS economics in China, enriches the data for veterinary economics, and re-stresses the necessity for producers and veterinarians to control PRRS effectively.

## Introduction

Porcine reproductive and respiratory syndrome virus (PRRSV) is an enveloped positive-strand RNA virus and the causative agent of PRRS, which was first identified in Europe in 1991 and the United States in 1992 (1, 2). Recently, PRRSV was reclassified to the genus *Betaarterivirus* of the family *Arteriviridae* in the order *Nidovirales*, consisting of two species, PRRSV 1 (European) and PRRSV 2 (North American) based on the identity at the nucleotide level (International Committee on Taxonomy of Viruses, ICTV) (3). The clinical symptoms of PRRS depend on the strain of PRRSV and the age of infected pigs, characterized by massive reproductive failure in sows (increased abortion rates, decreased litter size, etc) and severe respiratory disorders in growing pigs (higher mortality, increased health costs, etc) (4–6). In addition to the pathogenicity of PRRSV isolates, herd status, various management practices, and other factors can influence the clinical manifestation of PRRS (7, 8). A lot of studies were conducted to explore the economic effect of the PRRS outbreak and huge losses have been proven during the past 30 years, although the severity and, consequently, the financial losses after PRRSV infection vary greatly. At the national level, Neumann et al. (9) estimated and reported that swine producers lose nearly $561 million each year in the US, but a more recent calculation in 2013 estimated the cost of productivity losses due to PRRS to be as high as $664 million per year (10). At the individual farm level, Nathues et al. (8) presented a model and determined the annual losses ranged from a median of €75,724 to a median of €650,090 in Switzerland. For outbreak cases, early research from 1992 reported that the cost of an acute PRRS outbreak in four PRRSV negative herds in Illinois was $100, $170, $428, and $510, with a mean of $302 per breeding female (10). A Dutch study from 2012 in seven regular commercial production and two nucleus herds showed the economic losses varied between €59 and €379 per sow per 18-week period PRRS outbreak (11). A Spanish study calculated the losses at $200 per sow or $17.7 per slaughter pig on a farrow-to-finish farm and $122 per sow or $13 per piglet (12 kg live weight) on a breeding farm (8). Finally, the costs associated with persistent infections of PRRSV (endemic PRRSV infection) in 21 German sow herds were analyzed ranging from €46 to €568 per sow and year (12).

More than two decades have passed since the first PRRS report in the 1990s, accompanied by several epidemics due to the broad variation and rapid evolution of PRRSV in China (5, 13). Especially in 2006, a highly pathogenic PRRSV (HP-PRRSV), originating from a CH-1a-like virus, emerged with high fever, high morbidity, and high mortality (14, 15). The introduced NADC30-like and recombinant strains between the NADC30-like and local Chinese strains have been dominant in the field since 2014, with various clinical manifestations (16, 17). In the last 2 years, a rising NADC34-like PRRSV has been detected and isolated in some regions of China (18, 19). However, despite the terrible impact of PRRS on the Chinese swine industry, there was no available economic analysis or disease impact assessment of PRRS. In our previous study, we systematically analyzed the main metrics affected by PRRS outbreaks in four cases (20), the objective of this study was to estimate the economic losses of the four outbreaks based on the production deficiencies.

## Method

The methodology of our analysis is a relatively simple and direct approach modified from the economic model used by Nieuwenhuis et al. (11), which was only focused on the additional costs and decreased revenues. The gross margin, revenue, and saving costs were not analyzed in this study. The additional costs (the increased vaccine and medication costs, etc) and decreased revenues (the decreased numbers of weaned piglets, etc) due to the PRRS-induced production deficiencies were calculated based on the average cost-of-production indices from annual estimates of costs between 2014 and 2017 (Supplementary Table 1). The price of feed and the different ages of pigs were acquired from the website: http://www.boyar.cn/. Co-infection with bacteria can exacerbate PRRS-associated disease, but these data were difficult to accurately collect, and costs were difficult to apportion among multiple infectious agents, so the multiple secondary infectious diseases, such as *Haemophilus parasuis* and *Streptococcus suis*, that impacted production for the fattening herds were excluded from the study.

### The information of the four PRRS outbreaks

Three breeding farms (A, B, C) with the sale of piglets at weaning and one farrow-to-finish farm (D) from a large-scale swine company were selected to participate in this study, with the average number of sows of 5,000, 2,450, 3,800, and 1,750. The PRRS outbreak occurred in four breeding herds in November 2014, March 2015, December 2016, and February 2017, respectively, based on clinical signs discovered by veterinarians and diagnosis and confirmed by laboratory tests, with PCR negative results for classical swine fever virus (CSFV), pseudorabies virus (PRV), and porcine circovirus 2 (PCV2) in the detected samples. Four different PRRSV strains, SDwh1403 (a recombinant betweenNADC30-like and MLV), SDqd1501 (a recombinant between HP-PRRSV-like and QYYZ), SDwh1601 (a recombinant between JXA1-P80 (a MLV derived from HP-PRRSV and NADC30-like), and SDwh1701 (a strain evolved from JXA1-P80) were isolated (21). All four herds were vaccinated with the PRRS modified live vaccine (MLV) three times per year before the outbreak. At the time of the PRRS outbreak, the four farms adopted the “load-close-exposure” method by introducing native gilts that will be needed for replacement during the next 5–6 months. The two herds, A and B, were vaccinated with MLV once and the other two herds were exposed to the specific (farm) strain of PRRSV (sera acclimatization), and then all were vaccinated with MLV in intervals of 4 weeks. The information was summarized in Supplementary Table 2. The outbreak of PRRS was considered terminated when the veterinarian claimed the clinical manifestations (stillbirths per litter, mummies per litter and abortion rate, etc.) had disappeared and the productivity had returned to basic performance (the number of weaned piglets).

### The calculation of economic losses based on the production deficiencies

The main production data was acquired from the management system, 6 months before the PRRS outbreak to the basic production performance after the outbreak, to analyze the specific production parameters. Then, the calculation of economic losses was applied independently for breeding and fattening herds based on the specific comparative production metrics affected by PRRS. Representative costs derived from the average market prices reported during the period from 2014 to 2017 were generated to reflect typical historical costs in the industry. Variable cost standards in this study included ¥4,000/sow, ¥1,637/gilt, ¥380/weaned pig, ¥15.65/kg (marketed pigs), ¥3.217/kg of feed for sows, ¥2.928/kg of feed for gilts, ¥3.693/kg of feed for nursery pigs, and ¥2.752/kg of feed for grower-finisher pigs. The average cost of feed from wean to finish was ¥2.874/kg (about 35kg feed for nursery pigs and 235kg feed for finisher pigs) (Supplementary Table 1). The fixed costs of production were excluded from the study.

The economic losses of breeding herds mainly consist of (1) the increased vaccine and medication costs (including pre-weaning piglets); (2) the decreased numbers of weaned piglets * ¥380/weaned pig; (3) the increased numbers of eliminated sows * ¥4,000/sow; and (4) the increased numbers of replaced gilts * ¥1,637/gilt. The water, electricity, and increased labor costs were assumed not to be affected by PRRS in breeding herds and were not calculated in this analysis. The costs due to the low quality of weaned piglets (positive for qRT-PCR) were analyzed in fattening herds.

The economic losses of fattening herds mainly consisted of (1) increased vaccine and medication costs; (2) increased labor costs attributable to the delay to market (¥1.30/pig/day, including the equipment, water, electricity, and so on); (3) increased feed costs; (4) increased costs attributable to the increased mortality and cull rate [including labor costs (¥80/pig) and weaned-pig prices (¥380/pig)].

The economic losses of fattening herds = the marketed pigs * (the increased health cost /pig + the delayed marked days * ¥1.3 + the additional FCR * 115 kg * ¥2.874/kg)+(the weaned piglets * the additional mortality rate) * ¥460.

## Results

The four breeding herds took 20, 13, 18, and 17 weeks, respectively, to regain basic production performance, with a reduction in the number of weaned piglets and an increase in health costs. The average number of weaned piglets (per week) of herds A, B, C, and D in the 6 months before the PRRS outbreak was 2,166, 1,157, 1,815, and 868, and decreased to 1,933, 954, 1,487, and 723 after the outbreak, respectively. This represented a reduction of 4,660, 2,639, 5,904, and 2,465 pigs when they come back to the basic production performance, leading to 1.77, 1.00, 2.24, and 0.94 million losses based on the value of ¥380/weaned pig. For sows, it was estimated that 64, 39, 97, and 53 pigs were additionally culled than before, and an extra 158, 44, 159, and 92 gilts were introduced for replacement, which yielded losses of 0.52, 0.23, 0.65, and 0.36 million for the four herds, respectively. Furthermore, the increased vaccine and medicine costs (health costs) were also calculated for sows and piglets. Overall, the total losses in the four breeding herds were estimated to be ¥3.34, ¥1.65, ¥3.82, and ¥1.66 million, equal to an average of ¥822.75 per sow, ranging from ¥668.14 to ¥1004.43. The majority of the losses (56.92%) in the breeding herds were due to the reduction in revenue caused by decreased weaned piglets, and secondary losses were health costs with a proportion of 26.32%. The analyses of these parameters and losses are summarized in Tables 1, 2.

**Table 1:** The main economic parameters after the PRRS outbreak in the breeding herds.

**Parameters**	**Value**			
	**A**	**B**	**C**	**D**
Increased culled sows (head)	64	39	97	53
Increased introduced gilts (head)	158	44	159	92
Number of weaned piglets before the outbreak (per week)	2,166	1,157	1,815	868
Number of weaned piglets after the outbreak (per week)	1,933	954	1,487	723
Time to basic performance in breeding herds (week)	20	13	18	17
Decreased number of weaned piglets (head)	4,660	2,639	5,904	2,465

**Table 2:** The economic losses after the PRRS outbreak in the breeding herds.

**Parameters**	**Value (millions**, ¥**)**			
	**A**	**B**	**C**	**D**
Losses for increased health costs of sows	0.39	0.08	0.29	0.11
Losses for increased health costs of piglets	0.66	0.34	0.64	0.25
Losses for decreased number of weaned pigs	1.77	1.00	2.24	0.94
Losses for increased culled sows	0.26	0.16	0.39	0.21
Losses for increased introduced gilts	0.26	0.07	0.26	0.15
Total losses after the outbreak	3.34	1.65	3.82	1.66
Economic losses per sow (yuan, ¥)	668.14	672.56	1004.43	945.85

The economic impact of PRRS on fattening herds was calculated (Table 3) and mainly comprised of an increase in mortality rate and health costs, a reduction in feed efficiency, and average daily gain (ADG). After the outbreak, the breeding sows produced PRRSV-positive piglets. Nearly 42,364, 22,479, 33,500, and 16,379 weaned piglets were infected in the four herds, respectively, which represented an increase in mortality rate (2.85–9.58%), leading to the extra dead pigs (1,207, 2,154, 1,280, and 598 pigs) and increased labor costs. The numbers of marked finished pigs were 38,034, 19,060, 29,148, and 14,777, for the four herds with additional 1.24, 10.05, 6.03, and 5.82 days, respectively. The feed conversion ratio (FCR) for herds A, B, C, and D were 2.58, 2.61, 2.59, and 2.57%, respectively, and increased to 2.67, 2.73, 2.69, and 2.65 after the outbreak, bringing an additional ¥33.05 feed cost per finished pig. Furthermore, the increased health costs ranged from ¥4.21 to ¥15.76 per pig in the four herds. Based on the acquired parameters and formula, the total losses calculated in the four fattening herds were estimated to be ¥1.91, 2.30, 2.07, and 0.95 million, equal to an average of ¥601.62 per sow (¥318.64 to ¥937.14) and ¥76.49 per finished pig (¥50.17 to ¥120.46).

**Table 3:** The main economic parameters and economic losses (millions, ¥) after the PRRS outbreak in the fattening herds.

**Parameters**	**Value**			
	**A**	**B**	**C**	**D**
Sow herds (head)	5,000	2,450	3,800	1,750
The number of weaned piglets infected with PRRSV (head)	42,364	22,479	33,500	16,379
Increased mortality and cull rate (%)	2.85	9.58	3.82	3.65
Extra died finishers (head)	1,207	2,154	1,280	598
Market finishers (head)	38,034	19,060	29,148	14,777
Delayed marketing time (day)	1.24	10.05	6.03	5.82
Increased health costs per pig (¥)	4.21	15.76	9.77	11.83
Increased feed conversion ratio	0.09	0.12	0.1	0.08
Total losses after the outbreak (millions, ¥)	1.91	2.30	2.66	0.95
Economic losses per finisher (¥)	50.17	120.46	70.86	64.45
Economic losses per sow (¥)	381.64	937.14	543.50	544.20

Taken together, as shown in Table 4, the economic impact of PRRS on the whole herds varied between ¥1049.78 and ¥1609.70 per sow with a mean of ¥1424.37, comprising losses of ¥822.75 for sow herds (58.45%) and ¥601.62 for fattening herds (41.55%).

**Table 4:** The economic losses after the PRRS outbreak in the whole herds (per sow).

**Farm**	**Breeding herds losses (¥)/percentage (%)**	**Fattening herds losses (¥)/percentage (%)**	**Whole herds losses (¥)**
A	668.14/63.65	381.64/36.35	1049.78
B	672.56/41.78	937.14/58.22	1609.70
C	1004.43/64.89	543.50/35.11	1547.93
D	945.85/63.48	544.20/36.52	1490.05
Mean	822.75/58.45	601.62/41.55	1424.37

## Discussion

With few exceptions, PRRS has been considered to impose substantial economic losses, and PRRS economic studies have been conducted in the last century (9). Several models have been used to estimate the cost of PRRS, represented by enterprise budget and partial budget methods. Neumann et al. (9) and Holtkamp et al. (10) used an enterprise budgeting model to ascertain the cost of the disease at the animal level. The animal-level costs were then combined with information from the United States Department of Agriculture (USDA) and information on PRRSV infection rates and incidence of outbreaks at a national level from the survey of swine veterinarians to obtain an estimate of the annual cost of the disease to the US industry. Nieuwenhuis et al. (11) performed an economic analysis of the PRRS outbreak in nine sow herds by using a partial budget approach. Further, Nathues et al. (8) developed an epidemiological and economic model to perform a gross margin analysis and a partial budget analysis based on the changes in health and production parameters assumed for different PRRS disease severities at the individual farm level, which was modified and applied by Renken et al. (12) for determining the cost of “endemic” PRRSV infection in 21 pig herds in Germany. For all these methods used for estimating the losses, the results are generally based on the specific parameters that are most affected by PRRSV. To better understand the results of previous studies, it has to be kept in mind that the number of farms enrolled was limited and not randomly chosen, since inclusion depended on the manager's willingness to participate and provide the required data. In the present study, a more straightforward approach of directly focusing on the production metrics derived from previous studies was used to calculate additional costs and decreased revenues in PRRSV-affected populations by comparing the technical results of the outbreak period (time to the basic production performance, represented by the number of weaned piglets per week) with the data of a 6-month period preceding the outbreak. To our knowledge, this is the first report describing the economic losses of the PRRS outbreak in China. The strength of the study was that all four cases came from one company and all the productive data was acquired from the management system (not based on the literature and expert opinion), which provided more reliable and available information for analyzing the losses.

Among the four outbreaks, the calculated economic effect of the PRRS outbreak was ¥1424.37 per sow (¥1049.78–¥1609.70) from breeding to fattening, higher losses than the estimates of previous studies in other countries (8, 9, 12). However, it is difficult to make a rigorous comparison, since the health status of herds, vaccine and feed price, the market price for finishers, and other costs were variable among different countries. The structural analysis showed that over 58.45% (¥822.75 per sow) of the whole cost was derived from sow herds except for herd B with a severe secondary infection (a longer circulation of PRRSV in breeding herds arising from the repeated introduction), which was shown to be similar with the analyses of Neumann et al. (60.90%) and Holtkamp et al. (54.50%) (9, 10), suggesting that it would be more profitable to control PRRS in the breeding-farrowing phase. The same was found for sow herds in our study, where more than half the losses resulted from the reduction in the number of weaned piglets. One difference from previous studies (10, 12) is the increased feed cost, occupying 44.88%, was the primary source for fattening herds in our analyses, but not revenue foregone for the fewer sale of finished pigs. Linhares et al. (22) have reported the herds could achieve stability sooner after the outbreak if breeding sows had prior contact with PRRSV. The four outbreaks all started from breeding herds with a modified live virus (MLV) three times per year (a conventional health status), although their PRRSV status was not monitored and confirmed before the outbreak, suggesting bigger financial losses might be incurred by PRRSV-negative herds. The “load-close-exposure” method was applied in the four cases, which might be one reason for the interpretation of the results when it was compared with other cases (adopting different interventions). Different medication programs and immunization strategies (including a different MLV for piglets) were used for controlling the situation. As such, there were different health costs among the four breeding or fattening herds, but the efficiencies need to be further evaluated in the future as mentioned in previous studies (23, 24).

As mentioned previously, there was no gold standard to determine the economic losses attributable to the PRRS outbreak. An obvious limitation of this study was the exclusion of co-infected and secondary diseases impacting production, especially the potential porcine circovirus type 2 (PCV2) subclinical infection (25). The PCR results of PCV2 were accidentally positive at the farm level but there was no clinical manifestation associated with PCV2 in the four cases. Besides, the water, electricity, and labor costs were assumed not to be affected by PRRS in breeding herds and the use of PRRSV-free semen, modifying facilities, and implemented detection programs were all not included in the study. The time to stable (TTS) (four consecutive negative results for detecting PRRSV in weaning-age pigs) for sow herds was longer than the time to basic performance (TTBP) (20, 22, 26, 27), so the economic impact derived from our approach only provided a conservative estimation and the losses were higher than presented in our study. Too many factors, such as PRRSV strain, herd type, herd size, management level, health status, intervention strategies, and others, could influence the losses caused by a PRRS outbreak. Thus, the results of the present study could only be considered as a case study and do not represent the entire population of swine herds in China.

Our current study provides an exploration for evaluating the economic impact of PRRS in China. More analyses from different production systems or enterprises, at the herd level, and even at the national level, should be conducted for profitable intervention strategies and warranting economic input decisions in the future.

## Data availability statement

The original contributions presented in the study are included in the article/Supplementary material, further inquiries can be directed to the corresponding author/s.

## Ethics statement

Ethical review and approval was not required for the study of animals in accordance with the local legislation and institutional requirements. Written informed consent from owners of the animals was not required to participate in this study in accordance with the national legislation and the institutional requirements.

## Author contributions

ZZ and XQ interpreted the data and drafted the work. XQ co-ordinated the study. ZL, SY, and FR acquired the data from the management system used in the farms. TB, LS, and HL analyzed the data. BZ and LZ provided advice for the research analysis. All authors read and approved the final manuscript.

## Funding

This work was supported by the Natural Science Foundation of Jiangsu Province (BK20201005) and Nanjing Dr. Vet Health Management Co., Ltd.

## Conflict of interest

Authors ZL, SY, FR, and XQ were employed by the company Nanjing Dr. Vet Health Management Co., Ltd. The authors declare that this study received funding from Nanjing Dr. Vet Health Management Co., Ltd. The funder had the following involvement in the study: collection, analysis, and the decision to submit it for publication.

## Publisher's note

All claims expressed in this article are solely those of the authors and do not necessarily represent those of their affiliated organizations, or those of the publisher, the editors and the reviewers. Any product that may be evaluated in this article, or claim that may be made by its manufacturer, is not guaranteed or endorsed by the publisher.
