# Supplementary material for: The economic impact of porcine reproductive and respiratory syndrome outbreak in four Chinese farms: Based on cost and revenue analysis
Source: Front Vet Sci. 2022 Oct 12;9:1024720. doi: 10.3389/fvets.2022.1024720 (PMC9597626; doi:10.3389/fvets.2022.1024720)
Supplement: Supplementary file 1 [file Data_Sheet_1.docx]

**Supplementary data**

**Table S1. The referenced price used in the study.**

| Category | Parameters | 2014 | 2015 | 2016 | 2017 | Average |
| --- | --- | --- | --- | --- | --- | --- |
| Pig price | weaners（￥/head） | - | - | - | 380 | 380 |
|  | sows（￥/head） | - | - | - | 4000 | 4000 |
|  | market finishers（￥/kg） | 13.47 | 15.23 | 18.59 | 15.34 | 15.65 |
|  | gilts（￥/head） | 1350 | 1500 | 1900 | 1800 | 1637 |
| Feed price | gilts phase（￥/ton） | 3017 | 2924 | 2921 | 2848 | 2928 |
|  | finishers phase（￥/ton） | 2847 | 2736 | 2744 | 2680 | 2752 |
|  | nursery phase（￥/ton） | 3764 | 3722 | 3697 | 3589 | 3693 |
|  | sows phase（￥/ton） | 3298 | 3230 | 3212 | 3126 | 3217 |

- no data.

The price was from website: http://www.boyar.cn/.

Average feed cost (wean to finish) : (￥3.693/kg* 35kg+￥2.752/kg*235kg)/ (35kg+235kg)=￥2.874/kg.

**Table S2. The basic information of four porcine reproductive and respiratory syndrome virus (PRRSV) infected herds**

| Farm | Farm type | Herd size (sows) | Outbreak time | Vaccination strain^*^ | Isolated strain | Exposed virus | |
| --- | --- | --- | --- | --- | --- | --- | --- |
|  |  |  |  |  |  | gilts | breeding sows |
| A | two-point^#^ | 5100 | 2014.11 | TJM-F92 | SDwh1403 | Ingelvac+ Ingelvac | Ingelvac+ Ingelvac |
| B | two-point | 2450 | 2015.3 | Ingelvac | SDqd1501 |  |  |
| C | two-point | 3800 | 2016.12 | Ingelvac | SDwh1601 | FLV+ Ingelvac |  |
| D | farrow-to-finish | 1750 | 2017.2 | Ingelvac | SDwh1701 |  |  |

* TJM-F92 means a HP-PRRSV MLV strain, Ingelvac means IngelvacPRRS MLV.

^#^ “two-point” means the breeding farms with sale of piglets at weaning.
